# Integrative Analysis of Genome and Expression Profile Data Reveals the Genetic Mechanism of the Diabetic Pathogenesis in Goto Kakizaki (GK) Rats

**DOI:** 10.3389/fgene.2018.00724

**Published:** 2019-01-10

**Authors:** Yuhuan Meng, Ying Cui, Wenlu Zhang, Shuying Fu, Lizhen Huang, Hua Dong, Hongli Du

**Affiliations:** School of Biology and Biological Engineering – Department of Biomedical Engineering, South China University of Technology, Guangzhou, China

**Keywords:** diabetes, GK rat, genome, population genetic, putative artificial selective sweeps, glucose-stimulated insulin secretion, beta cell mass, insulin action

## Abstract

The Goto Kakizaki (GK) rats which can spontaneously develop type 2 diabetes (T2D), are generated by repeated inbreeding of Wistar rats with glucose intolerance. The glucose intolerance in GK rat is mainly attributed to the impairment in glucose-stimulated insulin secretion (GSIS). In addition, GK rat display a decrease in beta cell mass, and a change in insulin action. However, the genetic mechanism of these features remain unclear. In the present study, we analyzed the population variants of GK rats and control Wistar rats by whole genome sequencing and identified 1,839 and 1,333 specific amino acid changed (SAAC) genes in GK and Wistar rats, respectively. We also detected the putative artificial selective sweeps (PASS) regions in GK rat which were enriched with GK fixed variants and were under selected in the initial diabetic-driven derivation by homogeneity test with the fixed and polymorphic sites between GK and Wistar populations. Finally, we integrated the SAAC genes, PASS region genes and differentially expressed genes in GK pancreatic beta cells to reveal the genetic mechanism of the impairment in GSIS, a decrease in beta cell mass, and a change in insulin action in GK rat. The results showed that *Slc2a2* gene was related to impaired glucose transport and *Adcy3*, *Cacna1f*, *Bmp4*, *Fam3b*, and *Ptprn2* genes were related to Ca^2+^ channel dysfunction which may responsible for the impaired GSIS. The genes *Hnf4g*, *Bmp4*, and *Bad* were associated with beta cell development and may be responsible for a decrease in beta cell mass while genes *Ide*, *Ppp1r3c*, *Hdac9*, *Ghsr*, and *Gckr* may be responsible for the change in insulin action in GK rats. The overexpression or inhibition of *Bmp4*, *Fam3b*, *Ptprn2*, *Ide*, *Hnf4g*, and *Bad* has been reported to change the glucose tolerance in rodents. However, the genes *Bmp4*, *Fam3b*, and *Ptprn2* were found to be associated with diabetes in GK rats for the first time in the present study. Our findings provide a comprehensive genetic map of the abnormalities in GK genome which will be helpful in understand the underlying genetic mechanism of pathogenesis of diabetes in GK rats.

## Introduction

The number of people suffering from diabetes worldwide in 2017 was 451 million, with over one in four cases in China (121 million) (Cho et al., 2018). Among these people, more than 90% suffered from type 2 diabetes (T2D) which is characterized by hyperglycemia, insulin resistance, and insufficient insulin secretion. Being overweight and obese is strongly associated with T2D. However, T2D patients show considerably lower BMI value in Chinese or oriental population in comparison to the Western white population (Ma et al., 2014). Therefore, suitable animal models are needed to investigate the etiological and pathogenic mechanisms of T2D in the absence of obesity.

The Goto Kakizaki (GK) rat is a non-obese rodent T2D model with an average body weight 10–30% less than that of age-matched control Wistar rat (Portha et al., 2001) and which displays the T2D characteristics such as hyperglycemia, beta cell defects, and insulin resistance. GK rats can spontaneously develop diabetes and were established by repeated inbreeding of Wistar rats selected at the upper limit of normal distribution for glucose tolerance (Goto, 1988). Genetic factor is fundamental in diabetogenic phenotype of GK rats as the embryos of GK rats transplanted into Wistar rats can develop diabetes (Chavey et al., 2013). The genome-wide genetic quantitative trait loci (QTL) analysis of GK rats revealed several loci that were related to diabetic phenotypes: Niddm1 located on chromosome 1 has a major effect on postprandial hyperglycemia, insulin release (Niddm1i region) and insulin action (Niddm1b region) (Galli et al., 1999); Niddm2 on chromosome 2 and Niddm3 on chromosome 10 affect both postprandial and fasting hyperglycemia; Niddm3 affects insulin release (Galli et al., 1996). However, the underlying genetic mechanisms associated with these loci are poorly understood.

It has been reported that the impaired glucose-stimulated insulin secretion (GSIS) in pancreatic beta cells is a major factor responsible for glucose intolerance in GK rats (Portha et al., 2012) in which the beta cell mass is reduced by 60% (Portha et al., 2001). The impairment in GSIS mainly results from the dysfunction in insulin secretion pathway (Jensen et al., 2008) such as that observed in beta cell GLUT2-null mouse (Guillam et al., 2000). Several other genes have also been reported to be associated with GSIS. Transgenic mice expressing *Bmp4* in beta cells enhanced impaired GSIS and glucose clearance (Goulley et al., 2007). Also, *Fam3b* overexpressing mice displayed decreased insulin secretion (Cao et al., 2005) and increased glucose intolerance (Robert-Cooperman et al., 2014). In contrast, glucose intolerance was improved in *Fam3b* knockout mice (Moak et al., 2014). *Ptprn2* knockout mice displayed a 35.5% decrease in insulin secretion by glucose stimulation (Cai et al., 2011). A recent study on gene and protein expression profile in pancreatic beta cells in GK rats revealed that the early impairment in GSIS in GK beta cells may be caused by the Warburg-like metabolic shift (Hou et al., 2017). This study also inferred that beta cell reduction may result from early defects in proliferation (Hou et al., 2017). The reduction in beta cell mass in GK rats may also be related to the development of pancreas or cell neogenesis (Portha et al., 2012). In addition, GK rats displayed insulin sensitivity in the first 3 weeks after birth and became insulin resistance after 8 weeks (Movassat et al., 2008). However, the genetic mechanism of these features including the impairment in GSIS, a decrease in beta cell mass and a change in insulin action in GK rats remain unexplored.

For a more comprehensive and effective analysis of the genetic mechanisms of T2D in GK rats, we re-sequenced the genome of 10 GK rats and 10 control Wistar rats with a high coverage. We combined with another genome sequence data of 1 Wistar and 2 GK rats form previous studies (Atanur et al., 2013; Liu et al., 2015) and identified the homozygote and heterozygote variants in GK and Wistar populations and sought out the putative artificial selective sweep (PASS) regions in GK rats which were enriched in GK fixed mutations and may harbor key mutations resulting from primary selection. Finally, we integrated GK specific amino acid changed (SAAC) genes, the PASS region genes, and pancreatic differential expression genes to explore the genetic mechanism of the impairment in GSIS, decrease in beta cell mass, and the change in insulin action of GK rat.

## Materials and Methods

### Genome Sequencing and Variants Calling

The genome sequencing of 10 GK rats and 10 Wistar rats (8 weeks old) were purchased from SLAC Laboratory Animal Co., Ltd. (Shanghai, China). All rats used in this study were same with our previous study (Meng et al., 2016), which were approved by the institutional review board of the Guangdong Key Laboratory of Laboratory Animals. All protocols were carried out in accordance with the approved guidelines of the Institutional Animal Care and Use Committee, Ethic Certificate No: IACUC2014029. Genomic DNA was isolated from liver tissues using TIANamp Genomic DNA Kit (Qiagen, cat #DP304-02) following the manufacturer’s instructions. The genome DNA sequencing libraries were constructed by Annoroad Co., Ltd., and were sequenced using the Illumina HiSeq 2500 platform. Sequencing generated 150 bp paired-end raw reads. Duplicate reads caused by base-calling and adaptor contamination, reads with >5% unidentified nucleotides and with a phred quality <20 for over 50% of the read length were removed from the raw reads.

Clean data was obtained for further mapping with the rat reference genome. The rat reference genome and its corresponding annotation documents were downloaded from the Ensembl database. Genome mapping was used BWA (Li and Durbin, 2010) with “mem -t 10” parameter. Samtools (v1.6) and Picard (v2.5) were used for sorting, assigning read group information containing library, lane and sample identity. GATK (version 3.7) (McKenna et al., 2010) was used to call SNVs and small indels after dealing indel realignment and base recalibration. All steps were guided by the best practices workflows for variant discovery analysis at GATK office web. The dbSNP of Rat used in this analysis was downloaded from NCBI. CNVs (copy number variants) were called from CNVnator (Abyzov et al., 2011) (v 0.3.3) by the sorted mapping bam files. We obtained the CNVs of each sample with the filter of q0 < 0.5, e-val1 < 0.05 and e-val2 < 0.05, the probability of RD values within the region to be in the tails of a Gaussian distribution describing frequencies of RD values in bins.

### Phylogenetic Analysis of 65 Rats

In order to explore the genome-wide evolutionary relationship and divergence among GK, Wistar, and other laboratory rat strains, we constructed a phylogenetic tree with a total of 65 rat strains (44 strains including GK/slc and GK/ox from previous studies, 20 stains in our study and 1 reference genome). The variants data of 42 rats were downloaded from RGD database. Since the information of variants position of the 42 strains was available for Rn5.0 assembly, UCSC LiftOver was used to retrieve the new coordinates in Rn6.0 assembly. Variants of GK/Slc and Wistar/Slc were reanalyzed, and reads were acquired from European Nucleotide Archive with the accession number of PRJEB6678 (Liu et al., 2015). SNVs that were homozygous and without missing data across the 64 rats samples were used to construct a neighbor-joining tree with MEGA6 (Tamura et al., 2013).

### Population Genetics Analysis With 12 GK and 11 Wistar Rats

In order to analyze the genetics of these two populations, GK/ox, GK/Slc, and Wistar/Slc were re-analyzed with the same setting described above. The sequencing reads of GK/ox were downloaded from European Nucleotide Archive database with the accession number of ERP002160 (Atanur et al., 2013). A VCF file for 12 GK rats and 11 Wistar rats was obtained for further population genome analysis. Variants sites at least covered by 10 GK rats and 9 Wistar rats were retained for population analysis. The purpose for that is to get comprehensive and confident population variants, and exclude the variant missing called in some individuals with no coverage. To test whether different filtering criteria had significant effects on the subsequent data analyses, we calculated the fixed variants density, Tajima’s D and genetic differentiation between GK and Wistar rats for non-overlapping 10-kb windows using VCFtools (Danecek et al., 2011).

### GK PASS Region Identification and Overlapping With Rat T2D QTLs

As GK rats were selectively bred with glucose intolerance, the fixed variants may be concentrated on some specific regions of the whole GK genome. Therefore, we assumed that PASS regions which enriched for GK fixed variants may have arisen during selective breeding, and those regions may be positively selected in the initial phenotype driven derivation of the GK rats. Thus, we identified the GK PASS regions using homogeneity test (Liu et al., 2014) with the fixed and polymorphic sites between GK and Wistar populations by using a sliding window approach (50 kb window sliding in 10-kb step size) for each window. In this study, we defined:

*P*_GK_= number of polymorphic sites in the 12 GK rat samples;
*P*_W_= number of polymorphic sites in the 11 Wistar rat samples;
*F*_GK_= number of fixed differences between GK rat and both Wistar rat and the reference genome;
*F*_W_= number of fixed differences between Wistar rat and both GK rat and the reference genome.

We performed a homogeneity test for the null hypothesis *P*_GK_/*F*_GK_= *P*_W_/*F*_W_, equivalent to *P*_GK_/*P*_W_ = *F*_GK_/*F*_W_. We used a Pearson’s chi-square test on the 2 × 2 contingency table (R v3.4 with fisher test). *P*-value with multiple test by Bonferroni correction (R v3.4 with p adjustment and Bonferroni method) less than 0.05 were set as significant. And the significant windows with *F*_GK_>>*F*_W_ were regarded as the GK PASS regions. The overlap windows were merged into a region by bedtools (v2.27.1) (Quinlan and Hall, 2010) with merge option.

The candidate QTLs were obtained by filtering with “GK rat” or “T2D” from the rat QTLs downloaded from the FTP of RGD (Shimoyama et al., 2015). The same candidate QTLs were merged with the coordinate of position exhibited in Rn6.0, and finally 109 candidate QTLs were remained. Overlaps between PASS region and the candidate QTLs were identified by bedtools (Quinlan and Hall, 2010) intersect function.

### SAAC Genes and Integrative Analysis

The fixed variants of GK or Wistar rats were annotated by ANNOVAR (Wang et al., 2010). The genes with variants in GK or Wistar rats were defined as GK or Wistar SAAC genes, respectively. DAVIAD 6.8 (Huang da et al., 2009) was used to interpret the KEGG pathway with the GK and Wistar SAAC genes. GO (gene ontology) enrichment was performed in http://www.geneontology.org/. The SAAC genes that were involved in the GSIS/release, insulin signaling or pancreas/beta cell development pathways were marked. Bedtools (Quinlan and Hall, 2010) with intersect option were used to process the SAAC genes location information bed file and PASS region bed file. The SAAC genes overlapped with GK PASS regions were defined as GK PASS region genes. The differentially expressed genes of GK pancreas beta cell in 4, 6, and 8 weeks were remaining. The mapping reads of each gene’s raw count of GK pancreas beta cell were obtained from the Supplementary Table S2 of previous study (Hou et al., 2017). Raw reads count of all genes were normalized to TPM (transcripts per kilobase of exon model per million mapped reads). The differentially expressed genes were processed with Bayes-regularized paired *t*-test by Cyber-T bayesreg.R (Kayala and Baldi, 2012), and acquired by FDR < 0.05. The marked GK SAAC genes which were overlapped with GK PASS or with differentially expressed in beta cell on the early stage (4–8 weeks) were selected as candidate genes.

## Results

### Genome Re-sequencing, Genetic Variation, and Phylogenetic Tree of GK and Wistar Rats

To obtain diabetogenic variations in GK rats, we performed a population genomic re-sequencing of 10 GK diabetic rats and 10 normal control Wistar rats. In total, we obtained ∼744 billion bases of genomic re-sequencing data including two deep re-sequencing with average depth of ∼28× coverage (one GK and one Wistar rat) and 18 normal deep re-sequencing with average depth of 11× (nine GK and nine Wistar rats) (Supplementary Table S1). After genome mapping, variants calling and quality control processes, we obtained a total of 97.44% of mapping rate and identified 76 million high-quality single nucleotide variants (SNVs). The SNV homozygote rate for each rat was 91–93%. The detailed statistics of SNV, Indel and CNV of each rats listed in Table 1.

**Table 1:** The mapping information and variants in individual.

Sample	Clean base	Mapping rate %	Average depth	SNV-Homo	SNV-Hete	SNV-Homo rate	INDEL	CNV-dup	CNV-del
GK34	76706671800	97.37	27.57	7025904	644324	91.60%	2720477	2076	7763
GK35	33305105100	97.36	11.97	7007430	563611	92.56%	2460660	4447	3774
GK36	32378405400	97.42	11.64	7001861	554881	92.66%	2422592	5315	3663
GK38	32885229600	97.35	11.82	7003758	568359	92.49%	2435927	4654	3841
GK39	32538309600	97.42	11.70	6992229	541654	92.81%	2396650	6153	3505
GK54	33086972100	97.33	11.89	7011999	574173	92.43%	2468456	1690	5571
GK55	32005178700	97.40	11.50	7000243	573746	92.42%	2454205	3351	4654
GK56	32728872000	97.45	11.76	7006338	554427	92.67%	2432972	5317	4651
GK58	32722738200	97.40	11.76	6995459	552561	92.68%	2433417	6573	3940
GK59	32108272800	97.32	11.54	7001961	550865	92.71%	2423978	5837	4143
W33	78023244600	97.50	28.05	7197843	645283	91.77%	2547611	3572	3603
W35	30806483100	97.53	11.07	7150377	535719	93.03%	2226274	6916	3892
W37	31249128900	97.46	11.23	7173018	559716	92.76%	2254090	4545	3862
W38	30737268900	97.51	11.05	7156273	558375	92.76%	2251869	5883	3455
W39	32827799100	97.53	11.80	7174357	570610	92.63%	2299950	2898	3182
W52	32524719600	97.53	11.69	7176612	557664	92.79%	2277477	5505	4574
W54	32443915800	97.39	11.66	7156288	607150	92.18%	2299674	1094	5783
W55	32822133300	97.44	11.80	7167495	562180	92.73%	2273133	6337	3609
W56	32935132200	97.48	11.84	7177792	566297	92.69%	2291201	4880	3800
W59	32081037600	97.53	11.53	7146643	525204	93.15%	2222324	4294	1917

**FIGURE 1:**
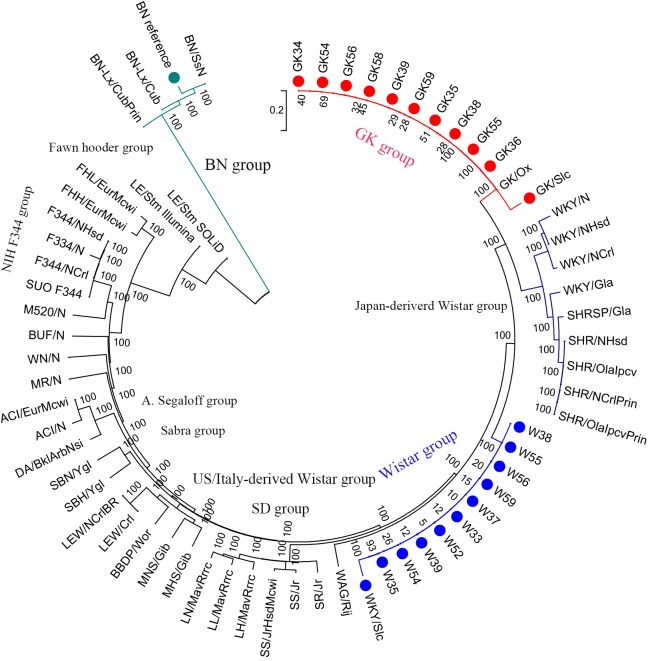
Phylogenetic tree of laboratory rats. The phylogenetic tree was constructed using 2.1 million homozygote SNVs exists in a total of 65 laboratory rat strains (including the BN reference strain in the green group) by MEGA6 with 100 bootstraps.

For the phylogenetic tree analysis, 10 GK rats were grouped with GK/slc and GK/ox, and 10 Wistar rats were clustered with Wistar/slc. Both groups were separated from the other Wistar-derived strains (Figure 1). Based on these results, a total of 12 GK rats (10 GK rats sequenced in the present study, 1 GK/Slc and 1 GK/ox) were set as one population, and 11 Wistar rats (10 Wistar rats sequenced in present study and 1 Wistar/slc) were set as the other population for further analysis.

### Population Variants and PASS Region in GK Rat

In order to get comprehensive and confident population variants, variants sites in 10 GK rats and 9 Wistar rats were retained for population analysis. In the two designated populations, there were a total of 6,939,902 and 6,157,702 variants in GK and Wistar rats, respectively. The number of the homozygote sites was 4,630,121 in GK rats, and 4,124,565 in Wistar rats. The homozygote rate was up to 91–93% in each sample but reduced to ∼67% at the population level (Table 1), indicating that the level of individual differentiation in these rats was high. A total of 2,915,315 homozygote sites were consistent with both GK and Wistar rats (over 60%), leaving 1,714,806 (37.06%) and 1,209,250 (29.32%) fixed variants in GK and Wistar rats, respectively. Among the fixed variants, 9,889 (0.578%) and 6,959 (0.575%) variants were located in the exonic regions in GK and Wistar rats, respectively.

**FIGURE 2:**
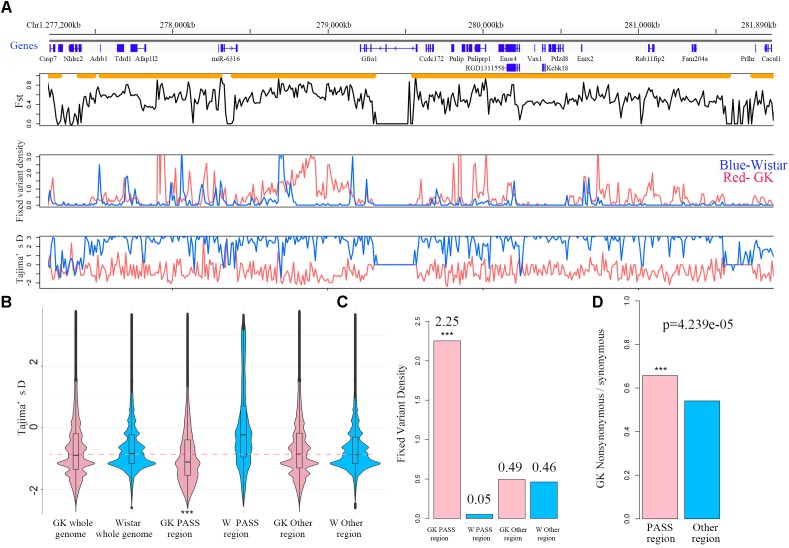
PASS regions in GK rats. **(A)** An example of the PASS region in GK rats. The gene distribution and the PASS regions (orange horizontal line) of the PASS region on chr1. Plot of the mean Fst in non-overlapping 10 kb between GK and Wistar rat across the PASS region. Distribution of fixed variant density and Tajima’D in non-overlapping 10 kb windows between GK (red line) and Wistar (blue line) rat in the examples of the PASS region. **(B)** Box plot of Tajima’D in non-overlapping 10 kb of whole genome, PASS and other regions between GK (pink) and Wistar (blue) rats. **(C)** The fixed variants density (variants count per 1 kb) of GK (pink bar) and Wistar (blue bar) rats in GK PASS region and other regions. **(D)** Ratio of the non-synonymous/synonymous of the PASS regions (pink bar) and other regions (blue bar) across the whole genome in GK rat. ^∗∗∗^*p* < 0.001.

For the PASS region identification, a total of 1,919 regions (segment sizes ranging from 50 KB to 1.5 MB, Supplementary Table S2) with approximately 190 MB (6.85% of whole genome) were identified as GK PASS regions. There were 2,095 coding genes, 98 miRNAs, 1,689 lncRNAs, and 258 other ncRNAs in the GK PASS regions. A detailed information about the distribution on each chromosome is provided in Table 2 and an example of PASS regions is shown in Figure 2A. To test whether the PASS regions were under artificial selection in GK rats, we performed the Tajima’s D test of PASS regions and other regions in GK and Wistar rats. The results showed that the Tajima’s D in GK PASS regions was lower than that in Wistar rats and other non-PASS regions in GK and Wistar rats (Figure 2B), indicating that the PASS regions in GK rats had undergone a strong selection. The GK fixed variants in GK PASS regions were 628,688 (36.67% in all GK fixed variants). In contrast, the Wistar fixed variants were only 9,648 (0.80% in all Wistar fixed variants). As shown in Figure 2C, the fixed variants density (variants count per 1 KB) in GK and Wistar rats in PASS regions and other non-PASS regions, demonstrated that GK fixed variants were significantly enriched in the PASS regions. In addition, 2,646 fixed variants occurred in coding regions of 923 GK genes in the PASS regions. However, only 92 fixed variants were present in coding regions of 54 Wistar genes in PASS regions which was too small to further estimate. Next, we estimated the ratio of non-synonymous coding variants to synonymous coding variants in GK PASS regions and other regions across the whole genome and found it to be 0.66 (1021/1553) and 0.54 (2481/4587), respectively (Fisher’s exact test *p* = 4.239e-05, Figure 2D), indicating that coding regions within the GK PASS regions were also under a selection. The GK PASS regions revealed a non-randomly distributed selective region in GK rats which may contribute to the hitchhiking effect of the key variants and may be associated with phenotypes in GK rats.

**Table 2:** The information of the GK PASS region.

Chr	PASS region (Mb)	Chr size (Mb)	PASS% in chromosome	PASS% in genome	Coding gene	miRNA	lncRNA	Other ncRNA
1	26.66	282.76	9.43%	0.96%	357	11	263	37
2	6.63	266.44	2.49%	0.24%	45	2	34	7
3	24.43	177.70	13.75%	0.88%	291	13	228	36
4	14.47	184.23	7.85%	0.52%	136	5	82	23
5	8.96	173.71	5.16%	0.32%	120	11	94	9
6	8.53	147.99	5.76%	0.31%	98	2	90	11
7	9.66	145.73	6.63%	0.35%	93	4	77	14
8	9.72	133.31	7.29%	0.35%	118	10	101	18
9	7.24	122.10	5.93%	0.26%	62	3	57	5
10	6.82	112.63	6.06%	0.25%	146	9	98	7
11	6.66	90.46	7.36%	0.24%	51	3	44	11
12	2.35	52.72	4.46%	0.08%	37	1	26	2
13	15.70	114.03	13.77%	0.57%	103	6	91	18
14	1.89	115.49	1.64%	0.07%	10	1	8	0
15	9.99	111.25	8.98%	0.36%	83	2	89	11
16	4.60	90.67	5.07%	0.17%	39	0	28	5
17	3.22	90.84	3.54%	0.12%	41	0	32	5
18	9.11	88.20	10.33%	0.33%	102	8	109	13
19	6.02	62.28	9.67%	0.22%	62	5	68	14
20	5.50	56.21	9.79%	0.20%	75	0	59	10
X	2.12	159.97	1.33%	0.08%	26	2	11	2
Total	190.28	2778.70	6.85%	6.85%	2095	98	1689	258

In the present study, we sought 109 candidate QTLs related to T2D or GK rats from RGD. Overall, 127 MB of 190 MB GK PASS regions (67%) overlapped with 101 of 109 candidate QTLs indicating most of the PASS regions were involved in GK or diabetes physiological phenotypes such as blood/plasma glucose level, body weight, serum insulin level, and pancreatic islet damage (Supplementary Table S3). The remaining non-overlapping PASS regions with candidate QTL may be related to the incomplete QTL research or the non-diabetes loci just introduced by the ancestor mutations from the process of repeated inbreeding.

### The SAAC Genes and Integrative Analysis Revealed the Genetic Mechanism of Pathogenesis of Diabetes in GK Rats

**FIGURE 3:**
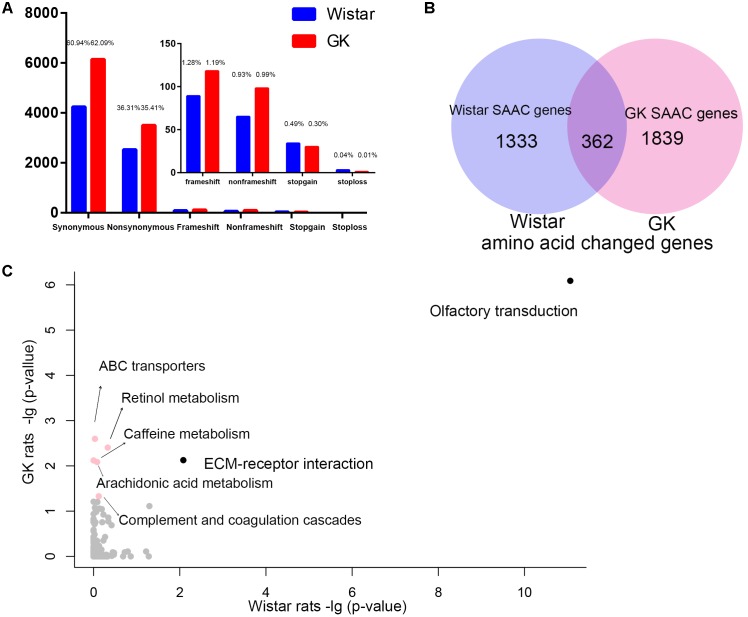
The exonic region fixed variants and the amino acid changed genes in GK and Wistar rats. **(A)** Distribution of synonymous, non-synonymous, and deleterious fixed variants (frameshift, non-frameshift, stopgain and stoploss) of the fixed homozygote variants in the exonic region of GK (red) and Wistar (blue), respectively. **(B)** Venn diagram of GK and Wistar amino acid changed genes. The GK/Wistar SAAC genes mean that the gene only exists as a the amino acid changed gene in GK/Wistar rats. **(C)** Data represents the –log(*P*-value) of the enrichment pathways in GK and Wistar SAAC genes. Enrichment pathways with high confidence [–log(*P*-value) > 1.3, *p* < 0.05] in different colors.

We found that there were 9,889 and 6,959 fixed variants located in the exonic in GK and Wistar rats, respectively (Figure 3A). Among these, there were 3,502 non-synonymous and 247 deleterious (frameshift, non-frameshift, stopgain and stoploss) fixed variants which caused amino acid change in 2,201 GK genes. In Wistar rats, there were 2,527 non-synonymous and 191 deleterious fixed variants which led to amino acid change in 1,695 Wistar genes. Among these genes, we found 1,839 GK SAAC genes that only existed as amino acid changed in GK rat and 1,333 Wistar SAAC gene that only existed as amino acid changed in Wistar rat (Figure 3B). The KEGG pathway functional enrichment analysis of GK and Wistar SAAC genes is presented in Figure 3C and Supplementary Table S4. The results show that enriched pathways were significantly related to diabetes indicating that few SAAC genes are influenced by diabetes in GK rats. Most of the GK SAAC genes may play a neutral role in the progression of diabetes in GK rats. However, some GK specific variants are expected to be responsible for the diabetic phenotype and are reflected in some specific genes. Therefore, we integrated the GK SAAC genes, GK PASS region genes, and GK differentially expressed genes in beta cell (Hou et al., 2017) to reveal the genetic mechanism of pathogenesis of diabetes in GK rat.

#### Impairment in GSIS

**FIGURE 4:**
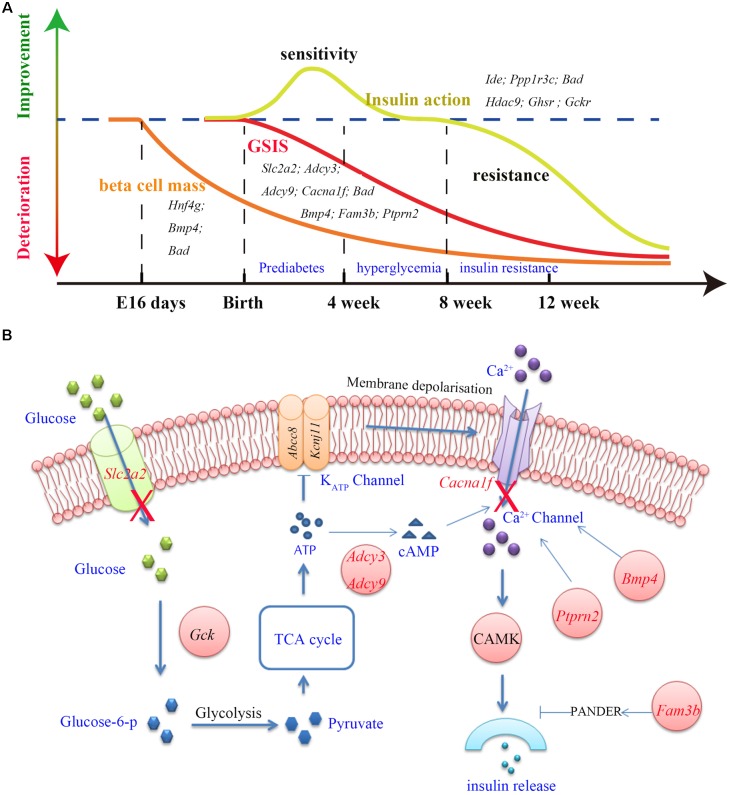
The three main features (GSIS, beta cell mass and insulin action) and insulin secretion pathway in GK rat. **(A)** The time course of expression of related genes associated with the GSIS (red), insulin action (yellow) and beta cell mass (orange) in GK rats. Impaired GSIS appeared early in the first week of life (Movassat et al., 2008); insulin sensitivity occurred in the first 3 weeks after birth (Movassat et al., 2008), and insulin resistance appeared in peripheral tissues at the age of 8 weeks (Portha et al., 2012); the beta cell mass reduction started as early as fetal age 16 days in GK rat (Miralles and Portha, 2001). **(B)** Related genes (red) involved in the insulin secretion pathway. In this pathway, glucose is transported into the beta cells by GLUT2 (coded by Slc2a2) and is phosphorylated to glucose-6-phosphate by glucokinase (Gck). Glucose-6-phosphate enters glycolysis and generates pyruvate. The TCA cycle then produces ATP and changes in the ATP/ADP ratio leading to the closure of K_ATP_ channel which in turn activates the Ca^2+^ channel by membrane depolarization which is required for insulin secretion. In addition, *Adcy3*, *Ptprn2*, *Bmp4*, and *Fam3b* genes were reported to be responsible for calcium transport and activity of Ca^2+^ channel.

One of the most distinguished diabetic features of GK rats is the impairment in GSIS. The impaired GSIS appears in the first week of life in GK rats (Movassat et al., 2008) (Figure 4A). Four genes (*Slc2a2*, *Adcy3*, *Adcy9*, and *Cacna1f*) related to the insulin secretion pathway were screened out in the present study. Among these genes, *Slc2a2*, which encodes the glucose transporter protein GLUT2, exists as a non-synonymous and a non-frameshift insertion in exon3. Besides, the mRNA and protein expression of *Slc2a2* was found to be significantly down regulated in GK rat beta cells (Table 3). Changes in the structural and expression of *Slc2a2* may influence glucose transport in GK pancreatic beta cells. In addition, *Adcy3* was found in the GK PASS regions and was differentially expressed in GK beta cells. The GK fixed mutations and down-regulation of *Adcy3* expression may influence the synthesis of cAMP and regulate insulin release in GK rats. Except for insulin secretion pathway, the other three GK SAAC genes (*Bmp4*, *Fam3b*, and *Ptprn2*) associated with GSIS were detected by integrating differential gene expression of GK pancreas beta cells. Therein, *Ptprn2* was identified as a GK PASS gene. The details of highly probable genes for GSIS in GK rats are provided in Figure 4B and Table 3.

**Table 3:** The detail of GK SAAC genes associate with GSIS, insulin action and beta cell mass.

Gene	Function	No. of S/N^a^	Amino acid change	Amino acid change effect	PASS region	Expression in beta cell (4–8 weeks)
*Slc2a2*	GSIS	3/2	G86R;W80	Benign;Probably damaging	No	Down
*Adcy3*	GSIS	4/1	L121P	Probably damaging	Yes	Down
*Adcy9*	GSIS	1/1	M145L	Benign	No	n.s^b^
*Cacna1f*	GSIS	3/2	R1631Q;T78S	Probably damaging;Benign	No	n.s
*Bmp4*	GSIS, beta cell mass	0/1	H207R	Benign	No	Down
*Fam3b*	GSIS	1/2	E197V;I145T	Benign;Benign	No	Up
*Ptprn2*	GSIS	2/3	A132V;R315H;V337A	Benign;Benign;Benign	Yes	Down
*Cela1*	Beta cell mass	2/2	R160K;P21A	Benign;Benign	No	Up/down
*Hnf4g*	Beta cell mass, insulin action	0/1	A134V	Benign	No	Down
*Dnaaf1*	Beta cell mass	0/1	H39R	Benign	Yes	n.s
*Bad*	Beta cell mass; GSIS, insulin action	0/1	Q92H	Benign	No	n.s
*Ide*	Insulin action; GSIS	1/2	A890V;H18R	Probably damaging;Benign	Yes	n.s
*Ppp1r3c*	Insulin action	3/1	A109V	Benign	Yes	n.s
*Hdac9*	Insulin action	1/1	E519D	Benign	No	n.s
*Ghsr*	Insulin action	0/1	V153E	Possibly damaging	No	n.s
*Gckr*	Insulin action	3/1	R149C	Possibly damaging	Yes	n.s

*^a^No. of S/N, the number of synonymous and non-synonymous variants. ^*b*^n.s, non-significant.*

#### Decrease in Beta Cell Mass

More than 50% reduction in beta cell mass was observed as early as fetal age of 16 days in GK rats (Miralles and Portha, 2001) (Figure 4A). In our study, five GK SAAC genes related to pancreas beta cell development, *Cela1*, *Hng4g*, *Bmp4*, *Dnaaf1* and *Bad* were identified from Gene Ontology database^1^. Among these genes, *Dnaaf1* was found in the GK PASS regions and *Bmp4*, *Cela1*, and *Hng4g* were detected with differential expression in GK beta cells in the early stage. The abnormalities in these genes may affect the pancreas or beta cell development which are likely reasons for the decrease in beta cells in GK rats. The variants information and expression of these genes is listed in Table 3.

#### The Change in Insulin Action

We found that insulin resistance occurred in peripheral tissues in GK rats at the age of 8 weeks. However, in the pre-diabetes period in GK rats (the first 3 weeks after birth), the whole body insulin sensitivity significantly increased when compared to the control Wistar rats (Movassat et al., 2008) (Figure 4A). The change in insulin action indicates that insulin resistance is not a primary diabetogenic reason in GK rats. Several GK SAAC genes have been previously reported to be involved in insulin action (listed in the Table 3). Importantly, *Ide*, *Ppp1r3c*, and *Bad* are located in Niddm1b, a diabetes susceptibility QTL which primarily affects insulin action and insulin-stimulated glucose transport (Galli et al., 1999; Fakhrai-Rad et al., 2000). As *Ide* and *Ppp1r3c* also belong to the GK PASS genes, they may associated with insulin sensitivity in GK rats. Moreover, *Hdac9* and *Ghsr* in adipose and *Gckr* (GK PASS gene) and *Bad* in liver were found to be associated with the insulin sensitivity. Interestingly, dysfunction or inhibition of some specific genes such as *Ide*, *Ppp1r3c*, *Hdac9*, *Ghsr* and *Gckr* can improve insulin sensitivity to varying degrees in pre-diabetes period in GK rats.

Among the highly probable genes involved in the pathogenesis of diabetes in GK rat, it has been demonstrated that the overexpression or inhibition of *Fam3b, Ptprn2, Ide, Hnf4g*, *Bad* and *Bmp4* is associated with the glucose tolerance in rodents (Supplementary Table S5). Here, we report for the first time that *Fam3b*, *Ptprn2* and *Bmp4* are related to diabetes in GK rats. The detailed variants and pancreatic expression of GSIS, beta cell mass, and insulin action related genes are listed in Table 3.

## Discussion

In this study, we constructed two population variants of 12 GK rats and 11 Wistar rats by whole genome sequencing and detected the GK PASS region and GK SAAC genes. The homozygote rate in each rat was 91–93% which was comparable to another study showing up to 96% homozygote rate in each sample (Liu et al., 2015). However, the homozygote rate in GK population with 12 GK rats was reduced to 67% suggesting that many variants did not exist in all GK rats. Therefore, the fixed variants detected in GK population can increase the accuracy and reduce noise variants. In addition, GK colonies at different locations exhibit different characteristics except for basal hyperglycemia (Portha et al., 2012) indicating that the newly introduced genetic variants are fixed in different GK colonies. The fixed variants based on population or different colonies (GK/Slc and GK/ox) in the present study may help us improve the targeted efficiency of diabetes-related loci. However, considering that the small sample size of 12 GK populations may influence the identification efficiency of diabetes relative genes in GK rat, we integrated the GK SAAC genes, GK PASS region genes, and differentially expressed genes in GK beta cells to reveal genetic mechanism of the impairment in GSIS, decrease in beta cell mass, and the change in insulin action in GK rat as they are the three major features of GK rats.

As GK rats are generated from repeated inbreeding of Wistar rats with glucose intolerance, the impairment in GSIS is regarded as the underlying cause of pathogenesis of glucose intolerance in GK rats (Portha et al., 2012). Normally, insulin released by beta cells is promoted when glucose is transported by GLUT2 protein and phosphorylated by glucokinase. The glucose-6-phosphate enters the glycolysis and increases ATP/ADP ratio which leads to the closure of K_ATP_ channels and further activates voltage dependent Ca^2+^ channels. The influx of calcium constitutes the triggering pathway required for insulin secretion. We found that the glucose transport was impaired and the Ca^2+^ channel was disordered in the insulin secretion pathway in GK rats. The non-synonymous and non-frameshift insertion variants in *Slc2a2* may change the structure of GLUT2, a glucose transporter in pancreatic beta cells. The down-regulation of *Slc2a2* expression in GK pancreatic beta cells (Hou et al., 2017), may influence the GSIS in GK rats. *Adcy3* belongs to the family of adenylyl cyclases which encodes enzymes that catalyzes the synthesis of cAMP, an important second messenger regulating insulin release. In human beings, loss of function variants of *ADCY3* can increase risk of obesity and T2D (Grarup et al., 2018). *Adcy3* has an amino acid changed in GK rat and shows a decreasing expression in GK pancreatic beta cell which indicates that *Adcy3* is a highly probable candidate gene responsible for impaired GSIS in GK rats. In addition to its regulatory role in beta cell growth and function (Bruun et al., 2014), *Bmp4* is also related to GSIS. The overexpression of *Bmp4* increased the GSIS in mice through *Bmp4-Bmpr1a* signaling (Goulley et al., 2007). In GK rats, *Bmp4* exhibits one non-synonymous mutation and is down-regulated in beta cells which may be associated with the impairment in GSIS. Deletion of *Ptprn2* in mice showed a significant decrease in GSIS, and a decrease in insulin secretion which may correlate with the decrease in the number of dense core vesicles and a change in Ca^2+^ channel activity (Atanur et al., 2013). The non-synonymous variants and decrease expression of *Ptprn2* in GK rats may alter the Ca^2+^ channel activity and influence the GSIS. *Fam3b*, also known as PANDER (pancreatic-derived factor), is a cytokine secreted in endocrine pancreas and plays a key role in beta cell function (Robert-Cooperman et al., 2010). Up-regulation of *Fam3b* inhibited insulin secretion by affecting intracellular Ca^2+^ homeostasis in beta cells (Cao et al., 2005). Moreover, *Fam3b* transgenic mouse overexpressing *Fam3b* exclusively in the endocrine pancreas exhibited glucose intolerance and insulin resistance (Bruun et al., 2014). In contrast, *Fam3b* knockout mice displayed enhanced metabolic phenotypes particularly enhanced glucose tolerance (Moak et al., 2014). In GK rats, the disorder of *Fam3b* (increased expression in pancreatic beta cell and two non-synonymous mutations) may be associated with the impairment in GSIS. In brief, the variations and dysregulated expression of *Slc2a2, Adcy3*, *Bmp4*, *Fam3b*, and *Ptprn2*, as well as the variations in genes *Adcy9* and *Cacna1f* may be implicated in the impaired GSIS in GK rats.

In pre-diabetes period, the whole body insulin sensitivity of GK rats increased significantly when compared to Wistar rats but the rats became insulin resistance at 8 weeks (Portha et al., 2012). This change implies a presence of compensatory mechanisms in GK rats. *Ide*, *Ppp1r3c*, *Hdac9*, *Ghsr* and *Gckr* were related to insulin action in GK rat. Interestingly, function-degrading or ablation of these genes could enhance the insulin sensitivity under certain conditions. Acute *Ide* inhibition leads to a substantial improvement in glucose tolerance in response to oral glucose administration (Maianti et al., 2014). The two non-synonymous variants (H18R and A890V) in GK IDE protein contribute to reduced insulin-degrading activity by 31% (Fakhrai-Rad et al., 2000) which suggests that the GK fixed mutations in *Ide* may act as a compensatory molecule to protect GK from diabetes. The homozygous *Ppp1r3c* ablated mice displayed reduced glucose and insulin levels after long-term high fat diet (HFD) reflecting an increased insulin sensitivity by the way of reducing hepatic glucose output (Lu et al., 2014); glucose tolerance and insulin sensitivity were found to be improved in *Hdac9* genetic ablated mice (Chatterjee et al., 2014) suggesting the anti-diabetes function of *Ppp1r3c* or *Hdac9* ablation. Besides, ablation of *Ghsr* can reduce adiposity and improve insulin sensitivity during aging by regulating fat metabolism in white and brown adipose tissues (Lin et al., 2011). In addition, the *Ghsr* knockout mice fed with HFD can improve insulin sensitivity by suppressing adipose inflammation, reducing macrophage infiltration in adipose tissue (Yuan et al., 2018). Moreover, *Gckr* encodes protein GKRP which plays a the key role in hepatic glucose and lipid metabolism via inhibition of glucokinase (Raimondo et al., 2015). Suppressing *Gckr* by small molecule inhibitors of GKRP could reduce blood glucose levels in rodent models of diabetes without short-term side effects on insulin or lipids (Lloyd et al., 2013). Therefore, the GK SAAC genes *Ide*, *Ppp1r3c*, *Hdac9*, *Ghsr* and *Gckr* with their specific mutations, may change the protein functions and improve insulin sensitivity in the prediabetes stage in GK rats and act as anti-diabetics in GK rats. However, insulin action occurred in peripheral tissues, the expression and variants information how to influence of those genes were insufficient in current. Therefore, further investigations of these genes is needed to better understand their roles in GK rat.

In the present study, we detected the population variants, GK PASS regions, and GK SAAC genes by whole genome sequencing of diabetic GK and control Wistar rats. In addition, we integrated the GK SAAC genes, PASS region genes, and differentially expressed genes in GK pancreatic beta cells to reveal the genetic mechanism of the impairment in GSIS, decrease in beta cell mass, and the change in insulin action in GK rats. Our study discovers novel genetic mechanisms of pathogenesis of diabetes in GK rats which may be helpful in better understanding the progression of diabetes.

## Data Availability Statement

The sequencing reads were available in NCBI SRA database (https://www.ncbi.nlm.nih.gov/bioproject/PRJNA479378/).

## Author Contributions

HDu conceived the original idea. YM, WZ, and SF performed the experiments. YM and YC carried out the data analysis and wrote the main body of the manuscript. LH, HDo, and HDu supervised the whole work, attended the discussions, and revised the manuscript.

## Conflict of Interest Statement

The authors declare that the research was conducted in the absence of any commercial or financial relationships that could be construed as a potential conflict of interest.

## Footnotes

1 http://www.geneontology.org/

## Supplementary Material

TABLE S1 The statistics of sequence reads and mapping information.

TABLE S2 The coordinates of PASS region.

TABLE S3 The information of PASS regions overlap with QTLs related to GK rat or T2D.

TABLE S4 The GK and Wistar SAAC genes and the KEGG functional enrichment.

TABLE S5 The GK SAAC genes related to the change of glucose tolerance.
